# Effectiveness of Shock-Absorbing Insole for High-Heeled Shoes on Gait: Randomized Controlled Trials

**DOI:** 10.3390/healthcare10101864

**Published:** 2022-09-24

**Authors:** Yu-Jin Cha

**Affiliations:** Department of Occupational Therapy, Semyung University, Jecheon 27136, Korea; occujin@semyung.ac.kr

**Keywords:** high-heeled shoes, surface electromyography (EMG), plantar pressure, randomized controlled trial, shock-absorbing insole

## Abstract

This study was carried out to identify the influence of a shock-absorbing insole, developed by the author for use with high-heeled shoes, on walking. The research design included single-blind randomized parallel groups; namely, a group of 26 participants who wore the shock-absorbing insoles and another group of 26 participants who did not wear the insoles, both carried out walking while wearing 7 cm high-heels. During walking, plantar pressure analysis (via in-shoe plantar pressure measurements), surface electrode electromyography (surface EMG), gait analysis, subjective comfort evaluation, and functional movement (functional mobility) analysis were carried out. In order to compare the two groups, statistical verification (paired t-test) was performed. Wearing the shock-absorbing insole with the high-heeled shoes improved posture stability during walking, as well as increasing the walking speed. In addition, the heel pressure, the pressure of the front foot at the inner side, and the shock ability were decreased. For these reasons, the wearers reported higher comfort. Changes in the muscle activities of the tibialis anterior muscle (TA) and the gastrocnemius muscle (GA) heightened the stability of the ankle joints. Overall, the proposed shock-absorbing insole for use with high-heeled shoes improved the postural stability when walking, as well as improving the distribution of pressure on the soles. A decrease in the diverse side-effects of wearing high-heeled shoes can thus be expected.

## 1. Introduction

Despite extensive warnings from public health organizations and international medical societies regarding the hazards of high-heeled (HH) shoes, the population wearing them in everyday life remains high [1]. According to the change of the center of mass of the body, not only do HH shoes change the body alignment, they also have harmful influences on walking and the function of the lower extremities [2]. High external knee joint loads have been related to anterior cruciate ligament injury [3], lower extremity over-use injury [4], knee pain [5], and osteoarthritis of the knee [6].

In order to absorb the increased vertical shock inflicted when walking while wearing HH shoes, not only are biomechanical adaptations of the spine needed, but the kinematic and kinetic characteristics are also altered [7]. Therefore, an effective method for reducing the adverse effects of HH shoes should be provided. Insoles, for either decreasing the impact force related to HH shoes or for absorption, may be an important solution in this context. An insole that absorbs impact force can improve the sole pressure distribution while walking, and can effectively prevent leg injury [8]. Insoles that are inserted to improve the stability of the feet and the shoes, as well as the sense of wear, have diverse material and physical characteristics [9].

Regarding previous research—including studies on the influence of the use of in-soles on the forces between the foot and the ground and reducing the maximum impact force—the effect of the insole regarding the ground reaction force and the sole pressure has been reported [1,7,10]. To date, research on the effect of insoles, in terms of reducing the impact force while walking, and studies comparing the subjective comfort of consumers through the analysis of exercise dynamic data in actual circumstances of use have been inadequate.

Regarding the human body as a connected chain [11], a compensation action appears after wearing HH shoes. As a result, in this research, a method to offset the negative influence of wearing HH shoes is developed. Regarding a shock-absorbent insole for use with HH shoes developed by the researcher, in order to determine its influence on walking, the alleviation of shock absorption, along with the results of in-shoe plantar pressure, a surface EMG, a gait analysis, subjective comfort, and functional mobility measurements, are compared and analyzed.

## 2. Materials and Methods

The research protocol and the written consent were examined and approved by the Institutional Review Board at Semyung University, case number SMU-2021-04-003-01. With adult women aged from 20–50 as the subjects, the purpose and method of this research was explained prior to the study. The women who consented, in writing, to participation were selected. All of the experiments were carried out according to the Helsinki Declaration of the World Medical Association. From August 2021 until October 2021, 56 participants were recruited. The criteria for selecting the subjects of the research were as follows:

1. Healthy adult women;
2. Persons who consented to the purpose and the method of this research, which were explained to them beforehand;
3. Persons who had not suffered from a musculoskeletal injury to the lower extremities within the past year;
4. Persons who did not have an orthopedic disability or pain in the lower Extremities;
5. Persons whose buttocks, knees, and ankle joint range of motion (ROM) were in the normal range;
6. Persons who satisfied height in the range of 155–175 cm and WHO Standard Normal BMI Index of 18.5–24.9.

The height (cm) and weight (kg) of the subjects were measured using an automatic height measuring machine GL-150 (G-tech International, Uijeongbu, Korea), in an upright position and taking off their shoes. Body mass index (BMI) was calculated by measuring height (m) and weight (kg) and dividing the weight by the square of the height (kg/m^2^) [12]. Shoe size and International Physical Activity Questionnaires (IPAQ) were collected through a self-administered questionnaire.

### 2.1. Sample Size

Regarding the size of the research sample, by utilizing the G*Power 3.1 Program, as a result of calculating the effect size (d = 0.80), significance (⍺ = 0.05), and examination ability (1 − β = 0.80), the number of subjects was calculated as 52 persons in total, with 26 persons per each group. Taking into account the in-the-middle failure rate of the subjects of the research being 10%, although 28 persons were selected for the experiment group and 28 persons were selected for the control group (a total of 56 participants) [13,14], as there were four participants whose data were inadequate, the final number of subjects was 26 in the experimental group and 26 in the control group (for a total of 52 participants).

### 2.2. The Information Regarding the Shock-Absorbing Insole for Use with HH Shoes

**Figure 1:**
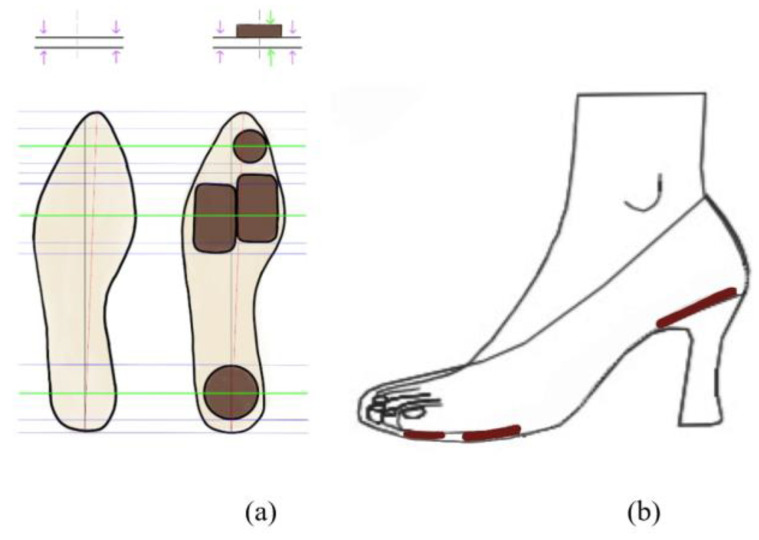
(**a**) The shock-absorbing insole for use with HH shoes; and (**b**) an illustration of the insertion of an insole in a high-heel shoe, where the height of the heel is 7 cm.

In order to prepare a plan for reducing the pressure concentrated on a specific part when wearing HH shoes, the researcher developed a shock-absorbing insole for use with HH shoes, which protects the body by reducing foot fatigue and dispersing the weight to the entire foot, based on the results of an analysis of the distribution of the plantar pressure and a biomechanical analysis. Regarding the shock-absorbing insoles for use with HH shoes, referring to the results of a previous experiment, in which a leading foot pressure analyzer was used, after the insoles were directly inserted into the shoes [15], the feet were protected from impact force and the feeling of the wearer was heightened. In particular, the hindfoot felt more cushioned by the insole. By increasing the entire thickness and by considering factors such as shock absorption, pressure dispersion, permeability, anti-bacterial characteristics, weight, stability, etc., the insole was developed. The special feature of the design of the insole product is the presence of cushions at four parts of the foot: The hallux, the medial forefoot, the lateral forefoot, and the heel. In addition, there is an adhesive sticker, making it is a product that can replace the standard cushion for shock relief, according to the shape of the foot, including a marking line for cutting to different sizes (Figure 1).

### 2.3. Study Design

**Figure 2:**
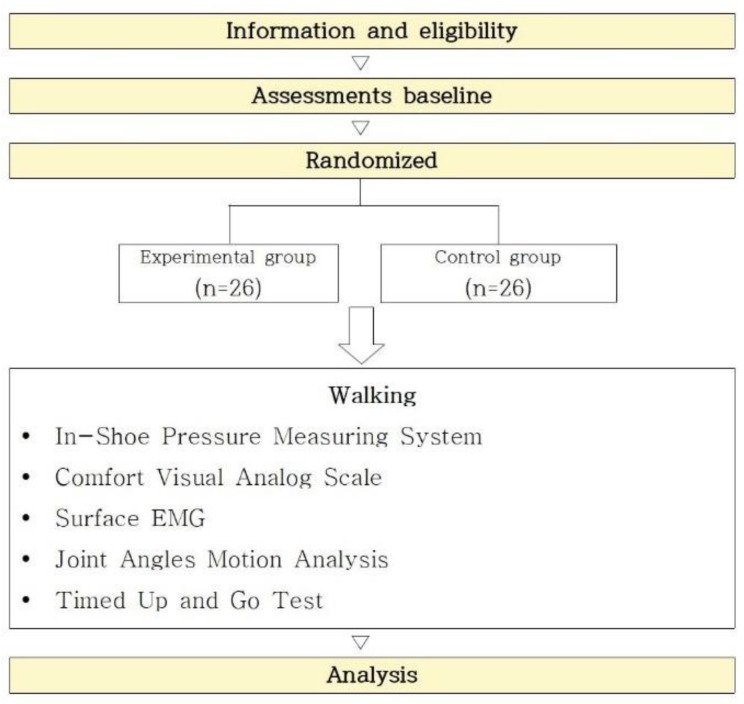
Protocol for a single-blind, randomized, parallel-group study.

Through randomization (specifically, a randomization table), the subjects were allocated to a group wearing the insoles (the experimental group) or a group that did not wear the insoles (the control group). When participants were assigned to a group randomly, they were not informed whether they were in the experimental group or control group. A flowchart explaining the experimental protocol is provided in Figure 2.

### 2.4. Experimental Procedure

In order to exclude environmental influences, all measurements were carried out in a measurement room where there was no noise. Regarding the area of the contact surface of the floor with the heels of the 7 cm high-heel shoes, both were around 1 cm^2^. To fit the sizes of the feet of all subjects, the same model product was applied. An EMG electrode was attached before measuring the plantar foot pressure. The activities of the anterior of the dominant side leg, the tibialis anterior (TA), the gastrocnemius (GA), the vastus lateralis (VL), and the biceps femoris (BF) that appeared while the subject walked were all measured. The measurement device and the variables used in this research are detailed in Table 1.

**Figure 3:**
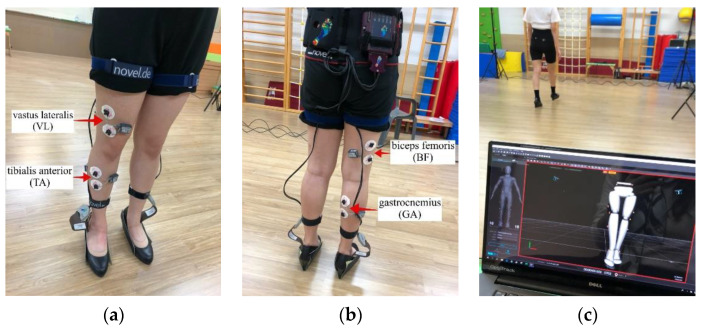
Scenes of the experiment: (**a**,**b**) System for measuring the pressure inside the Pedar-X shoes and the EMG electrode; and (**c**) the 3-dimensional movement analysis system (Optitrack 3D Motion Capture System).

At the same time, the sole pressure was measured using a system for measuring the pressure inside the Pedar-X shoes. An examination (Timed Up and Go Test; TUG) of walking for 3 m at a comfortable speed after standing up was performed (Figure 3a,b). Next, 16 markers were attached to the lower extremities. Kinematic data of the movement information, obtained through a three-dimensional movement analysis system (Optitrack 3D Motion Capture System), were measured as a 3-dimensional video. The collected data were stored in a connected computer (Figure 3c). In order to determine the dominant leg, the subjects were asked to kick a ball, and the leg they kicked with was decided as the dominant leg [16]. All of the measurements were carried out three times, with a rest interval of one minute. The average value of the three repetitions was used in the analysis. In order to reduce biases between examiners, one trained examiner performed all of the measurements.

**Figure 4:**
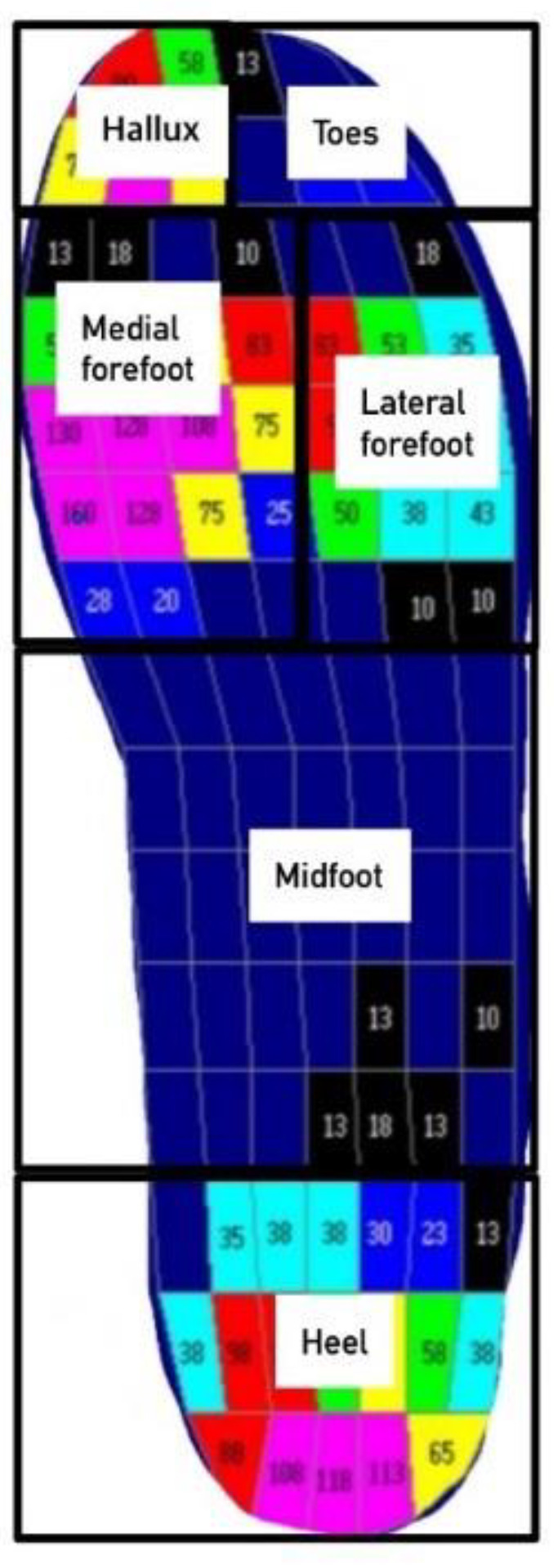
The six parts of the Pedar in-shoe pressure measurement system measurement.

In order to confirm the effects of the insole, a system (Novel Corporation, Munichen, Germany) for measuring the pressure inside the Pedar-X shoes was used, which incorporated a sensor and was connected to a data analyzer; in this way, the gait cycle, the stance period, and the swing period were analyzed. The measurements locations were at six parts of the sole (hallus, toes, medial forefoot, lateral forefoot, midfoot, and heel; see Figure 4). The peak pressure (PP), the contact area (CA), and the force time integral (FTI) were also analyzed. The FTI provides an understanding of the load distribution applied over time [17]. The system has been shown to be effective and reliable in measuring the pressure of the sole [18].

**Figure 5:**
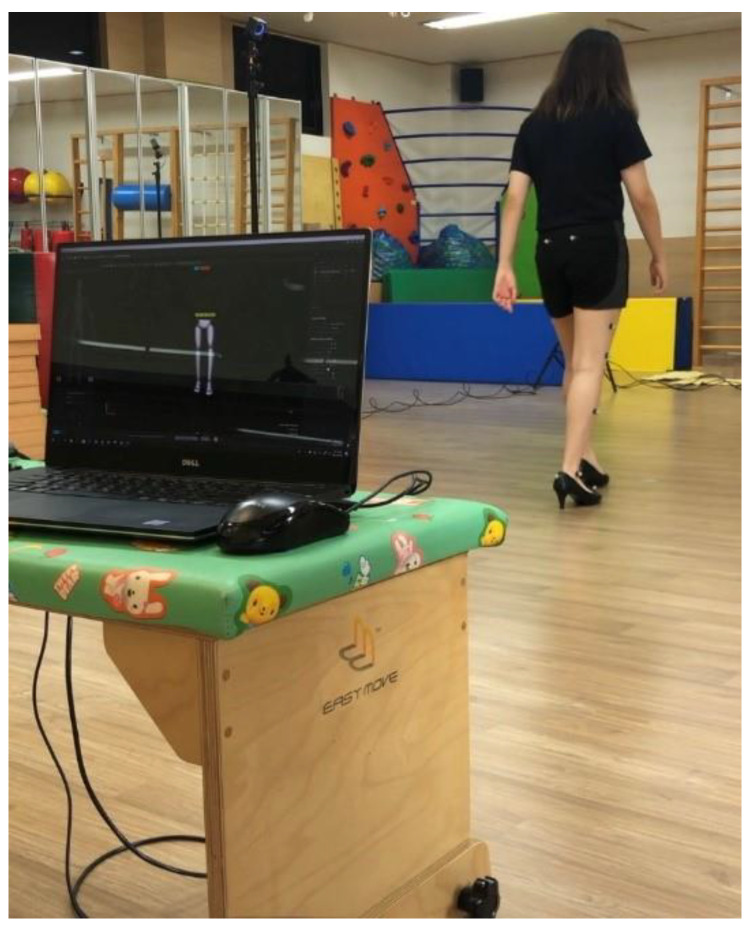
Reflection markers used for the 3-dimensional Movement Analysis System (The Optitrack 3D Motion Capture System).

For the electromyogram (EMG) measurement, an electromyogram system (Telemyo 2400T, Noraxon USA Inc., Scottsdale, AZ, USA) was used. Four agonistic muscles—the TA, the GA, the VL, and the bicepts femoris (BF)—among the lower extremity agonistic muscles that are mainly used when walking, were measured. Through repeated measurements of the dominant leg for a total of three times, the root mean square (RMS) EMG (uV) was collected [19]. After rectification of the radio waves of the EMG signals, RMS handling was applied. Regarding the EMG signals, a standardization process was carried out for comparison between the subjects and between the muscles. By using the muscular contraction in a specific movement as the standard contraction, the percent reference voluntary contraction (%RVC) method was used to standardize the EMG signals [20]. Using an infrared light-based 3-dimensional optical camera (Optitrack 3D motion capture system), which has high measurement resolution and small error, kinematic characteristics were measured and analyzed quantitatively and objectively. After attaching 16 reflection markers (retroreflective surface markers, 9.5 mm diameter) to the participants, which can be recognize by the optical camera to record the location of the measurement, walking was performed in the recognition space of the optical camera (Figure 5). By using the Visual 3D V5 Professional software of the C Motion company, then converting the data via MATLAB, quantitative analysis was carried out. The simultaneous effectiveness of the Optitrack Motion Capture System was calculated by using an ICC of 95% CI [21].

### 2.5. Data Analysis

All of the statistics in this research were handled using the SPSS/PC 19.0 software for Windows (SPSS, Chicago, IL, USA). Regarding the general and specific characteristics of the subjects of the research, through descriptive statistics, the average, standard deviation, percentile frequency, and homogeneity between the two groups were analyzed through Mann–Whitney U and Chi-square tests. The normality of data was examined in all groups using the Shapiro–Wilk test. The data did not follow a normal distribution, so non-parametric tests (Kruskal–Wallis test) were used to analyze inter-group differences and correlations between variables. In order to identify the influence of the shock-absorbing insole for use with HH shoes on walking, regarding the results of the in-shoe pressure measuring system, the Comfort Visual Analog Scale, surface EMG, joint angles motion analysis, and the TUG, descriptive statistics regarding the experimental group and the control group (e.g., average, standard deviation) were obtained, and an independent t-test was conducted. Additionally, the effect size (ES) was calculated using the Cohen’s d statistic. A p-value less than 0.05 was deemed to indicate a significant difference. In order to determine the relationship between the sole pressure variables and the results regarding the feeling when wearing the shoes, Pearson’s product moment correlation coefficient (Pearson’s correlation coefficient) was calculated.

## 3. Results

Regarding the subjects who participated in this research, there were a total of 52 women, with 26 participants in the experimental group and 26 participants in the control group. The average age was 25.38 ± 8.27 years old in the experimental group and 25.31 ± 7.01 years old in the control group. The average BMI was 21.75 ± 1.99 years old in the experimental group and 21.38 ± 2.26 years old in the control group. Regarding the international physical activity questionnaire, for the amount of activity, the low physical activity sub-group in the experimental group accounted for 38.46%; meanwhile, in the control group, the moderate physical activity sub-group was high (at 42.31%). There were no significant differences in age, body weight, height, body mass index (BMI), shoe size, and International Physical Activity Questionnaires (IPAQ) between the groups. The result of the timed up and go walking examination (TUG) was 8.13 ± 0.98 s for the experimental group and 9.13 ± 1.22 s for the control group, showing a significant difference (*p* < 0.05; Table 2).

**Figure 6:**
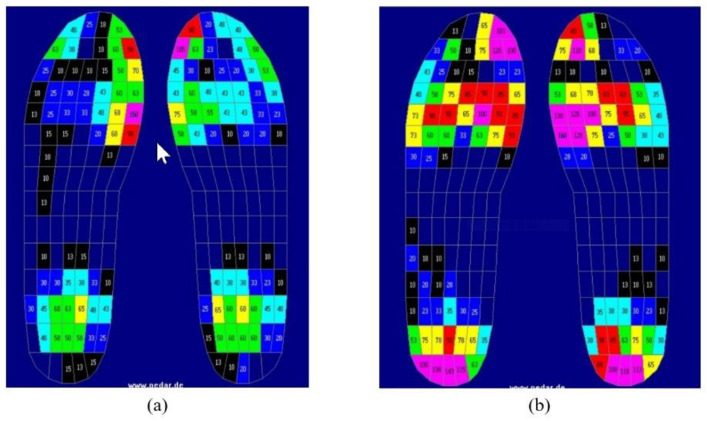
Examples of the plantar pressure distribution per region: (**a**) Wearing the insoles; and (**b**) not wearing the insoles.

Regarding the PP, in the three areas of the toes, the lateral forefoot, and the midfoot, there were significant differences between the group wearing the insoles and the group not wearing the insoles. Furthermore, the CA showed significant differences in the three areas of the medial forefoot, the lateral forefoot, and the midfoot. The force–time integral (FTI) showed significant differences in the six areas of the hallux, the toes, the medial forefoot, the lateral forefoot, the midfoot, and the heels. The ES was the largest for the FTI of the hallux (at 0.503), which denotes the greatest difference between the two groups. The red color in Figure 6 shows the regions with high pressure of the foot (i.e., high foot pressure). Compared to not wearing the insoles, the excessive foot pressure in the front foot area when wearing the insoles was reduced considerably. Overall, a uniform distribution of foot pressure can be observed with use of the insoles (Figure 6, Table 3).

**Figure 7:**
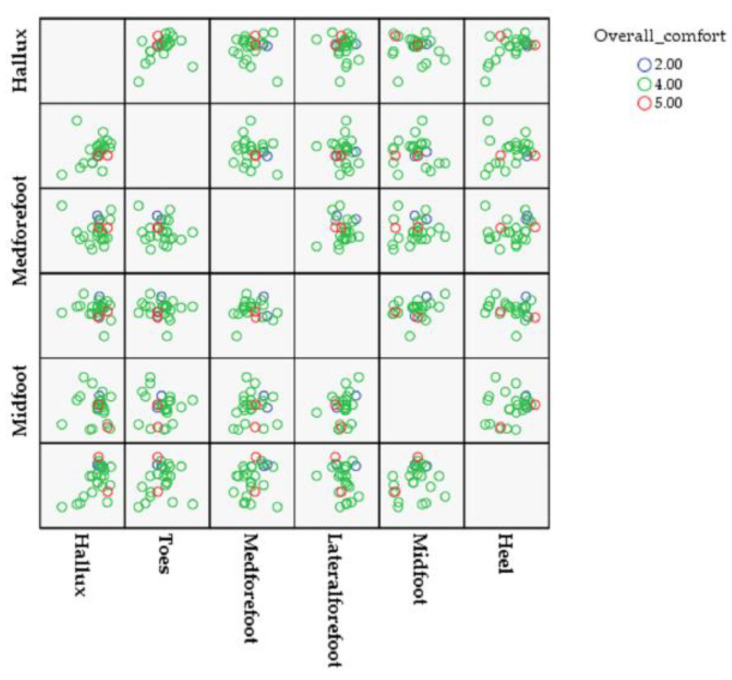
Pearson’s correlation coefficients between overall comfort and plantar foot pressure.

The results for the sole pressure variables, the feeling of wearing the shoes, and the correlations between the variables are shown in Figure 7 and Table 4. Regarding the correlation coefficients between the insole and the kinetic variables, in terms of the average PP value of the heel areas, a moderate positive correlation appeared (r = 0.555; *p* = 0.003).

The gait cycle showed significant differences in the VL, TA, and GA. For the stance period, the GA, the swing period, and the VL, significant differences were also observed. Regarding the %RVC, in the VL and in the GA, significant differences were shown. Regarding the gait cycle, the TA and the GA of the experimental group were more activated. For the stance period, the GA of the experimental group was more activated. For the swing period, the VL of the control group was more activated. Regarding the %RVC, the VL and the GA of the control group were more activated. The ES was the largest for gait cycle of GA (at 0.631), indicating the greatest difference between the two groups (Table 5).

**Figure 8:**
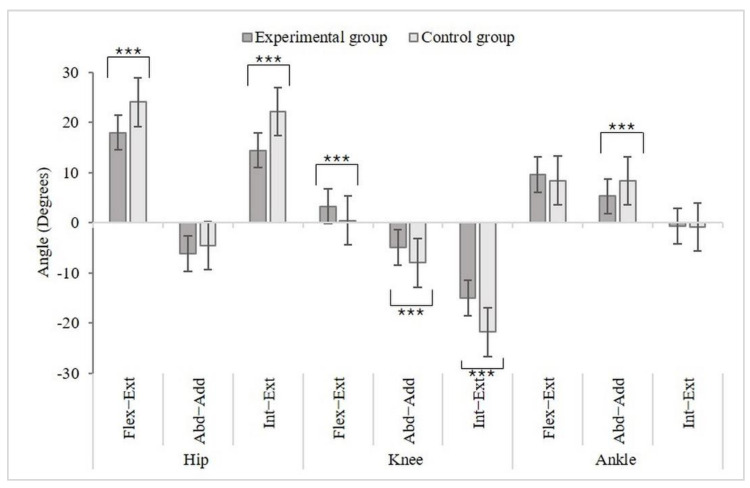
The joint angles for the hip, knee, and ankle during walking. ***, *p* < 0.001.

Regarding the hip joint, a significant difference between Flex-Ext and Int-Ext was observed. The control group displayed movement with a bigger angle. Regarding the knee joint, all three movements showed significant differences. In addition, the experimental group showed movements with larger angles at Flex-Ext and Abd-Add. In contrast, the control group showed movements with bigger angles at Int-Ext. Regarding the knee joint, Abd-Add in the control group showed movement with a bigger angle. The ES was moderate for flexion–extension of the hip, at 0.253 (Figure 8, Table 6).

**Figure 9:**
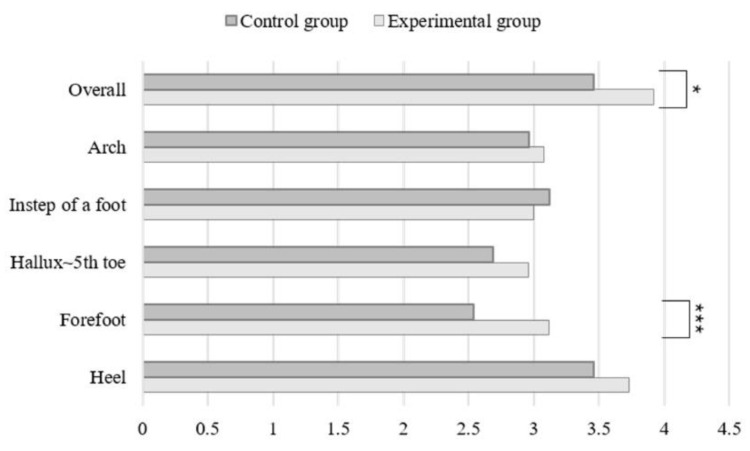
Comfort Visual Analog Scale (VAS) after walking. Statistical analysis by Kruskal–Wallis test; *, *p* < 0.05; ***, *p* < 0.001.

The results of a comfort test after walking are provided in Figure 9. For the overall feeling of wearing the shoes and at the forefoot, the comfort results for the group wearing the insoles were significantly higher (*p* < 0.05 and *p* < 0.001, respectively).

## 4. Discussion

Regarding the purpose of this research, through kinetic analysis of a shock-absorbing insole for use with HH shoes that effectively offsets the negative influence of wearing the HH shoes, in order to determine the influence on walking while alleviating the impact force through a re-distribution of the sole (plantar) pressure, the plantar pressure (in-shoe plantar pressure), surface EMG, gait, subjective comfort, and functional mobility were compared and analyzed.

Regarding the TUG—an examination that measures dynamic balance and movement capability [22]—the proposed shock-absorbing insoles for use with HH shoes improved the stability of the posture, compared to the case when not wearing the insoles. In addition, the walking speed increased. This is consistent with the result that insoles improve somatosensory function, and can be useful in alleviating age-based damage in the adjustment of balance [23]. Regarding the proposed shoe insoles, the mechanism by which the posture control is enhanced has been confirmed in previous research [24].

From the results of this research, regarding the FTI, the pressure of the group wearing the insoles decreased significantly in four areas—the hallux, toes, midfoot, and heels—relative to the group that did not wear the insoles. In contrast, regarding the two areas of the medial forefoot and the lateral forefoot, the pressure of the group wearing the insoles increased significantly, compared to the group not wearing the insoles. This was attributed to the shock-absorbing insole dispersing the pressure. The measurement results showed that the shock-absorbing insole considerably weakened the PP in the heels and the front foot parts. In addition, the increase in the area of contact of the midfoot domain to successfully redistribute the pressure was consistent with a study highlighting the important changes in the domain of the mid-foot [8].

The insole also reduced the heel pressure, the medial forefoot pressure, and impact force to the foot. This also coincided with a preceding study showing that insoles provide higher perceived comfort [7], as well as in agreement with a study reporting that, when insoles are used, the maximum impact force [25] and the loading rate [26] between the feet, the ground, and the knees may be effectively decreased. In addition, regarding individuals who have diverse pathologies (including knee pain), the efficacy of insoles in reducing the pain and the maximum impact force has been demonstrated in a previous study [27].

As a result of this research, regarding the feeling of wearing the shoes, the average pressure value and that in the heel part were high. Hence, in order to heighten the feeling of wearing the shoes, decreasing the pressure at the heel is important. If the insoles are used, together with sole pressure, as the impact force to the feet is decreased greatly, higher comfort can be perceived [1,25]. Regarding the VL, in the gait cycle, swing period, and %RVC, the group not wearing the insoles had higher activation than the group wearing the insoles. Regarding the TA and the GA, in the gait cycle, the group wearing the insoles was activated more than the group not wearing the insoles. When compared with the group not wearing the insoles, regarding the HH shoes, the %RVC of the VL and the GA was decreased. This coincided with a study reporting that the insole influences EMG activity in the lower extremities [6]. When wearing shock-absorbing insoles, due to increased ankle plantar flexion, in order to prevent a weight shift to the front, the TA muscle was activated more [28].

Furthermore, for the GA, the group not wearing the insoles showed higher activation. Regarding this, in a previous study, it has been reported that wearing high heels decreases stability through the imbalance of the muscles of the feet and the ankles by increasing the muscle activity of the GA. It was also reported that this can cause overall musculoskeletal system problems [29]. As a result, through activation changes of the TA muscle and the GA, by heightening the stabilities of the feet and the ankle joints, the walking pattern was improved. This could also prevent potential injury of the lower extremities.

The changes in the ankle dynamics and kinematics due to wearing HH shoes increase ankle instability and the danger of injury to the lower extremities [30]. From the results of this research, at the hip, the knee, and the ankle joint, the group not wearing the insoles showed movement with an overall larger angle, compared to the group wearing the insoles. Accordingly, wearing the shock-absorbing insoles improved the postural stability when walking. In addition, the pressure was dispersed, including the heel pressure and the medial front foot pressure, the impact force was decreased, and walking speed was increased. Due to this, higher comfort could be felt.

A limitation of this research is that it did not consider that the PP, the maximum strength, and the CA increased differently in specific domains, according to differences in the speed of movement of the subjects, although the participants were instructed to walk at their most comfortable walking speed. As a result, in future research, the speed of movement when walking (e.g., at comfortable walking speeds of 0.83–1.11 m/s) should be considered. Furthermore, in future work, the effect of the shock-absorbing insole should be verified not merely for walking, but also in other everyday life movements. Based on the results of this research, improvement of the awareness of the side-effects related to wearing HH shoes and the need for appropriate education for prevention should be emphasized.

## 5. Conclusions

The purpose of this research was to identify the influence of proposed shock-absorbing insoles for use with HH shoes on walking. To this end, the results of a plantar foot pressure analysis (via in-shoe plantar pressure), surface EMG, walking (gait) analysis, and subjective comfort and functional movement assessments were compared and analyzed. Wearing the shock-absorbing insoles improved the postural stability of participants when walking. In addition, their walking speed was increased. The heel pressure, medial front foot pressure, impact force experienced, etc., were also dispersed. For these reasons, higher comfort was reported. Through activation changes of the TA muscle and the GA, the stability of the ankle joint was heightened. In the future, the effect of the shock-absorbing insole for use with HH shoes should be verified not only when walking, but also in other everyday life movements.

## Figures and Tables

**Table 1:** The experimental equipment used to determine the influence of the designed insole on walking, as well as the shock absorption effect.

	Equipment Model (Company)	Variables	Unit
In-Shoe Pressure Measuring System	Pedar-X System (Novel Gmbh, Germany)	Peak pressure (PP) Contact area (CA) Force time integral (FTI)	kPa cm^2^ Ns/cm^2^
Comfort Visual Analog Scale (Comfort VAS)	-	comfort levels	score
Surface EMG	Noraxon EMG (Noraxon USA Inc., Scottsdale, AZ, USA)	Root Mean Square (RMS) Reference voluntary contraction (RVC)	uV %
Kinematics (Joint Angles) Motion Analysis	OptiTrack (Natural Point, OR, USA)	Degree	°
Timed up and go test (TUG)	-	functional mobility	sec

**Table 2:** Demographic characteristics of the participants (n = 52).

		Experimental Group (n= 26)	Control Group (n = 26)	*p*
Age (years)		25.38 ± 8.27	25.31 ± 7.01	0.973 a
Weight (kg)		57.70 ± 7.49	56.49 ± 8.24	0.598 a
Height (m)		162.62 ± 4.59	162.27 ± 6.26	0.793 a
BMI (kg/m^2^)		21.75 ± 1.99	21.38 ± 2.26	0.578 a
Shoe size		240.00 ± 5.10	239.42 ± 6.53	0.743 a
IPAQ	Low	11 (42.31) c	6 (23.08)	0.574 b
	Moderate	10 (38.46)	11 (42.31)	
	High	5 (19.23)	9 (34.62)	
TUG		8.13 ± 0.98	9.13 ± 1.22	0.004 ** a

**, *p* < 0.01; Values are presented as mean ± SD for continuous characteristics, and as percentages otherwise. BMI, body mass index; IPAQ, International Physical Activity Questionnaire; TUG, Timed Up and Go Test; a, p-value from Mann–Whitney U-test; b, p-value from Chi-square test; c, N (%).

**Table 3:** Plantar foot pressure in high-heel shoes during walking.

		Experimental Group (n = 26)			Control Group (n = 26)			*p*–Value	ES
		Mean	SD	95% CI	Mean	SD	95% CI		
Hallux	PP (kPa)	110.27	18.05	106.20–114.34	114.67	44.17	104.71–124.63	0.393	0.613
	CA (cm^2^)	8.99	17.08	5.14–12.84	6.26	1.29	5.97–6.55	0.164	0.099
	FTI (Ns/cm^2^)	184.75	53.85	172.61–196.90	209.16	58.56	195.96–222.36	0.005 **	0.503
Toes	PP (kPa)	41.38	14.66	38.08–44.69	50.34	12.86	47.44–53.24	0.000 ***	0.457
	CA (cm^2^)	5.60	5.35	4.40–6.81	5.87	1.55	5.52–6.22	0.678	0.197
	FTI (Ns/cm^2^)	76.07	35.46	68.07–84.06	121.42	38.15	122.82–130.02	0.000 ***	0.311
Medial forefoot	PP (kPa)	109.40	17.40	105.48–113.32	109.86	75.59	92.82–126.90	0.960	0.715
	CA (cm^2^)	13.97	18.44	9.81–18.12	9.21	2.91	8.55–9.86	0.028 *	0.216
	FTI (Ns/cm^2^)	441.60	105.91	417.72–465.48	363.85	97.14	341.95–385.75	0.000 ***	0.492
Lateral forefoot	PP (kPa)	75.94	13.27	72.95–78.93	59.07	43.09	49.35–68.78	0.002 *	0.346
	CA (cm^2^)	12.23	13.24	9.24–15.21	8.85	2.85	8.21–9.49	0.031 *	0.344
	FTI (Ns/cm^2^)	274.12	70.63	258.20–290.05	196.99	75.47	179.97–214.01	0.000 ***	0.430
Midfoot	PP (kPa)	12.20	6.37	10.76–13.63	15.79	8.03	13.98–17.60	0.002 **	0.354
	CA (cm^2^)	3.20	2.42	2.66–3.75	4.38	2.52	3.82–4.95	0.004 **	0.331
	FTI (Ns/cm^2^)	24.96	20.40	20.36–29.56	45.49	26.28	39.57–5.42	0.000 ***	0.297
Heel	PP (kPa)	76.12	22.06	71.14–81.09	86.57	49.19	75.48–97.66	0.106	0.511
	CA (cm^2^)	15.98	16.69	12.21–19.74	14.86	5.54	13.61–16.10	0.575	0.219
	FTI (Ns/cm^2^)	355.34	136.30	324.61–386.07	518.70	192.91	475.21–562.20	0.000 ***	0.478

*, *p* < 0.05; **, *p* < 0.01; ***, *p* < 0.001; FTI, force–time integral; MedFF, medial forefoot; CentFF, central forefoot; LatFF, lateral forefoot; ES, Effect Size; CI, 95% confidence interval (lower limit–upper limit); *p*-values from Kruskal–Wallis test.

**Table 4:** Pearson correlation coefficients (*r*) and p-values (*p*) for overall comfort and plantar foot pressure.

Variables	Overall Comfort of EG		Overall Comfort of CG	
	*r*	*p*	*r*	*p*
Hallux	0.045	0.829	0.382	0.054
Toes	0.337	0.093	−0.174	0.395
Medial forefoot	−0.082	0.691	0.385	0.052
Lateral forefoot	−0.131	0.525	−0.224	0.272
Midfoot	−0.096	0.64	−0.293	0.146
Heel	0.555 **	0.003	−0.049	0.813

**, *p* < 0.01; EG, Experimental group; CG, Control group.

**Table 5:** Muscle activity in high-heel shoes during walking. (unit: uV, %).

Type Event		Experimental Group			Control Group			*p*–Value	ES
		Mean	SD	95% CI	Mean	SD	95% CI		
Gait Cycle	VL	35.89	11.67	33.09–38.69	42.49	17.27	38.34–46.64	0.015 *	0.431
	TA	341.53	106.48	315.95–367.11	280.79	107.56	254.95–306.63	0.000 ***	0.472
	BF	458.69	172.93	417.15–500.24	496.54	158.51	458.46–534.62	0.179	0.367
	GA	712.65	282.33	787.86–883.81	835.83	199.70	644.82–780.47	0.002 **	0.631
Stances Period	VL	37.42	11.48	34.66–40.18	40.39	13.29	37.20–43.58	0.153	0.450
	TA	342.26	101.61	317.85–366.67	310.09	108.12	284.12–336.07	0.065	0.413
	BF	478.53	115.63	450.75–506.31	510.00	171.11	468.90–551.11	0.192	0.391
	GA	677.72	127.37	695.55–770.98	733.27	157.00	647.13–708.32	0.022 *	0.378
Swing Period	VL	28.53	11.53	25.76–31.30	43.11	14.06	39.73–46.49	0.000 ***	0.473
	TA	357.20	108.65	331.10–383.30	357.16	119.27	328.51–385.81	0.998	0.618
	BF	442.88	155.39	405.55–480.21	435.91	151.00	399.64–472.19	0.783	0.552
	GA	703.02	166.75	662.96–743.07	699.37	131.49	667.78–730.96	0.891	0.328
%RVC	VL	3.00	2.02	2.53–3.49	3.76	1.05	3.51–4.01	0.008 **	0.052
	TA	23.55	6.93	21.89–25.21	22.09	4.89	20.92–23.27	0.136	0.063
	BF	31.10	7.05	27.70–32.98	30.34	11.01	29.41–32.79	0.634	0.008
	GA	50.23	15.54	46.49,–53.96	55.91	15.73	52.13–59.68	0.038 *	0.118

*, *p* < 0.05; **, *p* < 0.01; ***, *p* < 0.001; TA, tibialis anterior; GA, gastrocnemius; VL, vastus lateralis; BF, biceps femoris; RVC, reference voluntary contraction; ES, Effect Size; CI, 95% confidence interval (lower limit–upper limit); *p*-values from Kruskal–Wallis test.

**Table 6:** The joint angles for the hip, knee, and ankle during walking (degree).

		Experimental Group			Control Group			*p*−Value	ES
		Mean	SD	95% CI	Mean	SD	95% CI		
Hip	Flex−Ext	3.28	6.43	1.83–4.73	0.42	2.54	−0.15−0.99	0.000 ***	0.253
	Abd−Add	−4.94	2.85	−5.58–4.30	−7.99	5.15	−9.15–6.83	0.055	0.319
	Int−Ext	−15.08	9.8	−17.29–12.87	−21.84	5.68	−23.12–20.56	0.000 ***	0.104
Knee	Flex−Ext	9.53	4.16	8.59−10.47	8.4	4.23	7.44−9.35	0.000 ***	0.017
	Abd−Add	5.25	2.97	4.58−5.92	8.34	4.6	7.31−9.38	0.000 ***	0.013
	Int−Ext	−0.64	2.65	−1.24–0.04	−0.89	2.3	−1.41–0.37	0.000 ***	0.003
Ankle	Flex−Ext	17.98	4.17	17.04−18.92	24.01	5.09	22.86−25.15	0.100	0.260
	Abd−Add	−6.22	3.76	−7.07–5.37	−4.61	6.26	−6.02–3.20	0.000 ***	0.198
	Int−Ext	14.44	9.97	12.19−16.68	22.15	5.18	20.99−23.32	0.486	0.158

***, *p* < 0.001; ES, Effect Size; CI, 95% confidence interval (lower limit–upper limit); *p*-values from Kruskal–Wallis test.

## Data Availability

Not applicable.

## References

[1] Wang M., Jiang C., Fekete G., Teo E., Gu Y. (2021). Health view to decrease negative effect of high heels wearing: A systemic review. Appl. Bionics Biomech..

[2] Moon G.S. (2014). The effect for the different height of high-heeled shoes on the lower extremity joint during the level running. Korean J. Sport Sci..

[3] Fox A.S. (2018). Change-of-direction biomechanics: Is what’s best for anterior cruciate ligament injury prevention also best for performance?. Sports Med..

[4] LaPlaca D.A., Seedman J. (2021). The importance of the foot and ankle in athletic performance. Strength Cond. J..

[5] Mishra E., Jena S., Bhoi C., Arunachalam T., Panda S.K. (2019). Effect of high heel gait on hip and knee-ankle-foot rollover characteristics while walking over inclined surfaces—a pilot study. Foot.

[6] Nguyen L.Y., Harris K.D., Morelli K.M., Tsai L.C. (2021). Increased knee flexion and varus moments during gait with high-heeled shoes: A systematic review and meta-analysis. Gait Posture.

[7] Liau Y.Y., Kim S., Jin S., Ryu K. (2021). The effect of wearing high-heels and carrying a backpack on trunk biomechanics. Int. J. Ind. Ergon..

[8] Gerych D., Tvrznik A., Prokesova E., Nemeckova Z., Jelen K. (2013). Analysis of peak pressure, maximal force, and contact area changes during walking and running with conventional and shock-absorbing insoles in the combat boots of the czech army. J. Mech. Med. Biol..

[9] Patwa R., Saha N., Sáha P. (2020). Magnetic hydrogel based shoe insoles for prevention of diabetic foot. J Magn. Magn. Mater..

[10] Kim B.G., Lee J.S., Yang J.O., Lee B.J. (2018). Analysis of the plantar pressure on the flat and slope walking by insole type. Korean J. Sport Sci..

[11] Charu G., Manoj M., Jaspreet K., Minaxi S. (2020). A systematic review and meta-analysis on effect of spinal mobilization and manipulation on cardiovascular responses. Hong Kong Physiother J..

[12] Kwon M.H., Han B.D., Cho S.J., Cho J.H. (2021). Analysis of body fat mass index for korean adults. Korean J. Fam. Med..

[13] Faul F., Erdfelder E., Lang A.G., Buchner A.G. (2007). G* power 3: A flexible statistical power analysis program for the social, behavioral, and biomedical sciences. Behav. Res. Methods.

[14] Párraga-Montilla J.A., Pozuelo-Carrascosa D.P., Carmona-Torres J.M., Laredo-Aguilera J.A., Cobo-Cuenca A.I., Latorre-Román P.Á. (2021). Gait performance as an indicator of cognitive deficit in older people. Gait Perform. Indic. Cogn. Deficit Older People.

[15] Cha Y.J. (2020). Analysis of differences in the degree of biomechanical adaptation according to habituation to different heel heights. Sci. World J..

[16] van Melick N., Meddeler B.M., Hoogeboom T.J., Nijhuis-van der Sanden M.W.G., van Cingel R.E.H. (2017). How to determine leg dominance: The agreement between self-reported and observed performance in healthy adults. PLoS ONE.

[17] Melai T., Ijzerman T.H., Schaper N.C., de Lange T.L.H., Willems P.J.B., Meijer K., Lieverse A.G., Savelberg H.H.C.M. (2011). Calculation of plantar pressure time integral, an alternative approach. Gait Posture.

[18] Price C., Parker D., Nester C. (2016). Validity and repeatability of three in-shoe pressure measurement systems. Validity Repeatability Three-Shoe Press. Meas. Syst..

[19] Elsais W.M., Preece S.J., Jones R.K., Herrington L. (2020). Between-day repeatability of lower limb emg measurement during running and walking. J. Electromyogr. Kinesiol..

[20] Kang J., Jeong D., Choi H. (2020). The effects of squat exercises with vertical whole-body vibration on the center of pressure and trunk muscle activity in patients with low back pain. J Int. Acad. Phys. Ther. Res..

[21] Dubois A., Bresciani J.-P. (2018). Validation of an ambient system for the measurement of gait parameters. J. Biomech..

[22] Ibrahim A., Singh D.K.A., Shahar S. (2017). ‘Timed up and go’ test: Age, gender and cognitive impairment stratified normative values of older adults. PLoS ONE.

[23] Aboutorabi A., Bahramizadeh M., Arazpour M., Fadayevatan R., Farahmand F., Curran S., Hutchins S.W. (2016). A systematic review of the effect of foot orthoses and shoe characteristics on balance in healthy older subjects. Prosthet. Orthot. Int..

[24] Losa Iglesias M.E., Becerro de Bengoa Vallejo R., Palacios Peña D. (2012). Impact of soft and hard insole density on postural stability in older adults. Geriatr. Nurs..

[25] Hong W.H., Lee Y.H., Lin Y.H., Tang S.F.T., Chen H.C. (2013). Effect of shoe heel height and total-contact insert on muscle loading and foot stability while walking. Foot Ankle Int..

[26] Nagano H., Begg R.K. (2018). Shoe-insole technology for injury prevention in walking. Sensors.

[27] Bonanno D.R., Landorf K.B., Munteanu S.E., Murley G.S., Menz H.B. (2017). Effectiveness of foot orthoses and shock-absorbing insoles for the prevention of injury: A systematic review and meta-analysis. Br. J. Sports Med..

[28] Yoo K.T. (2020). The effect of the insole height on lower limb joint angle and muscle activity at landing when the maximal ground reaction force of male in their 20s. J Converg. Inf. Technol..

[29] Hapsari V.D., Xiong S. (2016). Effects of high heeled shoes wearing experience and heel height on human standing balance and functional mobility. Ergonomics.

[30] Kim Y., Joo J., Jung J. Effects of custom-made 3D printed insoles on the trajectories of center of pressure of flat foot gait. Proceedings of the KSPE 2017 Spring Conference.

